# Does moving away from the center frequency resonance benefit or hurt the valvular structure evaluation? A study of *on* and *off* peak resonance

**DOI:** 10.1186/1532-429X-11-S1-T4

**Published:** 2009-01-28

**Authors:** Ronald B Williams, June Yamrozik, Vikas K Rathi, Geetha Rayarao, Robert WW Biederman, Mark Doyle

**Affiliations:** Allegheny General Hopital, Pittsburgh, PA USA

**Keywords:** Mitral Valve, Imaging Parameter, Structure Evaluation, Mitral Valve Leaflet, Valvular Regurgitation

## Introduction

Cardiac MRI (CMR) is the diagnostic modality of choice for ventricular volumes and function. The qualitative and quantitative valve function by CMR is performed by SSFP and phase contrast imaging respectively. However, often times the valve and subvalvular morphologic details are not clearly visualized in a reproducible fashion limiting the complete interrogation of the heart.

## Hypothesis

We propose that adjusting the center frequency resonance (CFR) as determined by the MRI interface for each sequence may, counterintuitively, produce optimal images for valve definition. By adjusting the center frequency manually to 'off' resonance, we hypothesize that CFR can exaggerate the susceptibility around the valves, making the valves easier to visualize.

## Methods

We scanned two healthy subjects without prior knowledge of their cardiac valvular structure or function. The imaging protocol was limited to the mitral valve and was performed on a 1.5 T GE HD Excite (Milwaukee, WI), 8 channel cardiac array coil; using the SSFP sequence. The imaging parameters were: matrix: 224/224; averages: 1; TR 3.285 (heart rate based); TE 1.42; Tdel, temporal resolution: 27 msec. (See Table 1.)

**Table 1:** Table 1

Subject 1					
Series	Resonance frequency (Hertz)	Transmit gain	R1 gain	R2 gain	Tuning method
Peak CFR	63868605	117	13	15	A
30° off Peak CFR	63868577	117	13	15	M
60° off Peak CFR	63858341	117	13	15	M
90 ° off Peak CFR	63868585	117	13	15	M
**Subject 2**					
Series	Resonance frequency (Hertz)	Transmit gain	R1 gain	R2 gain	Tuning method
Peak CFR	63868553	128	13	15	A
30° off Peak CFR	63868536	128	13	15	M
60° off Peak CFR	63868505	128	13	15	M
90 ° off Peak CFR	63868474	128	13	15	M

## Results

**Figure 1:**
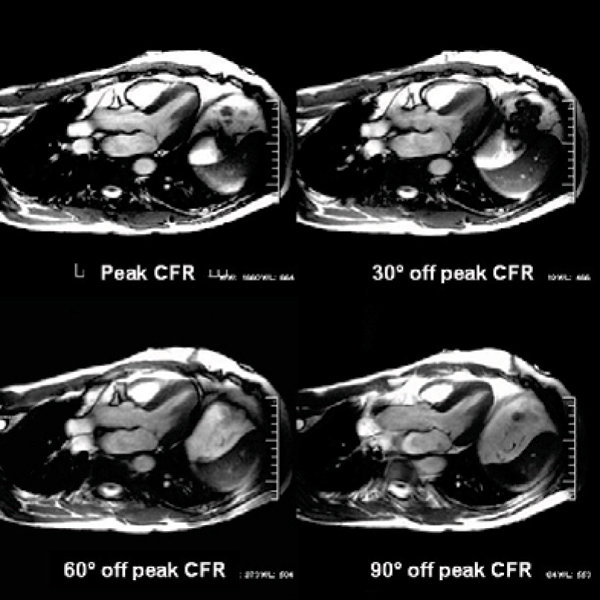
Figure 1

**Figure 2:**
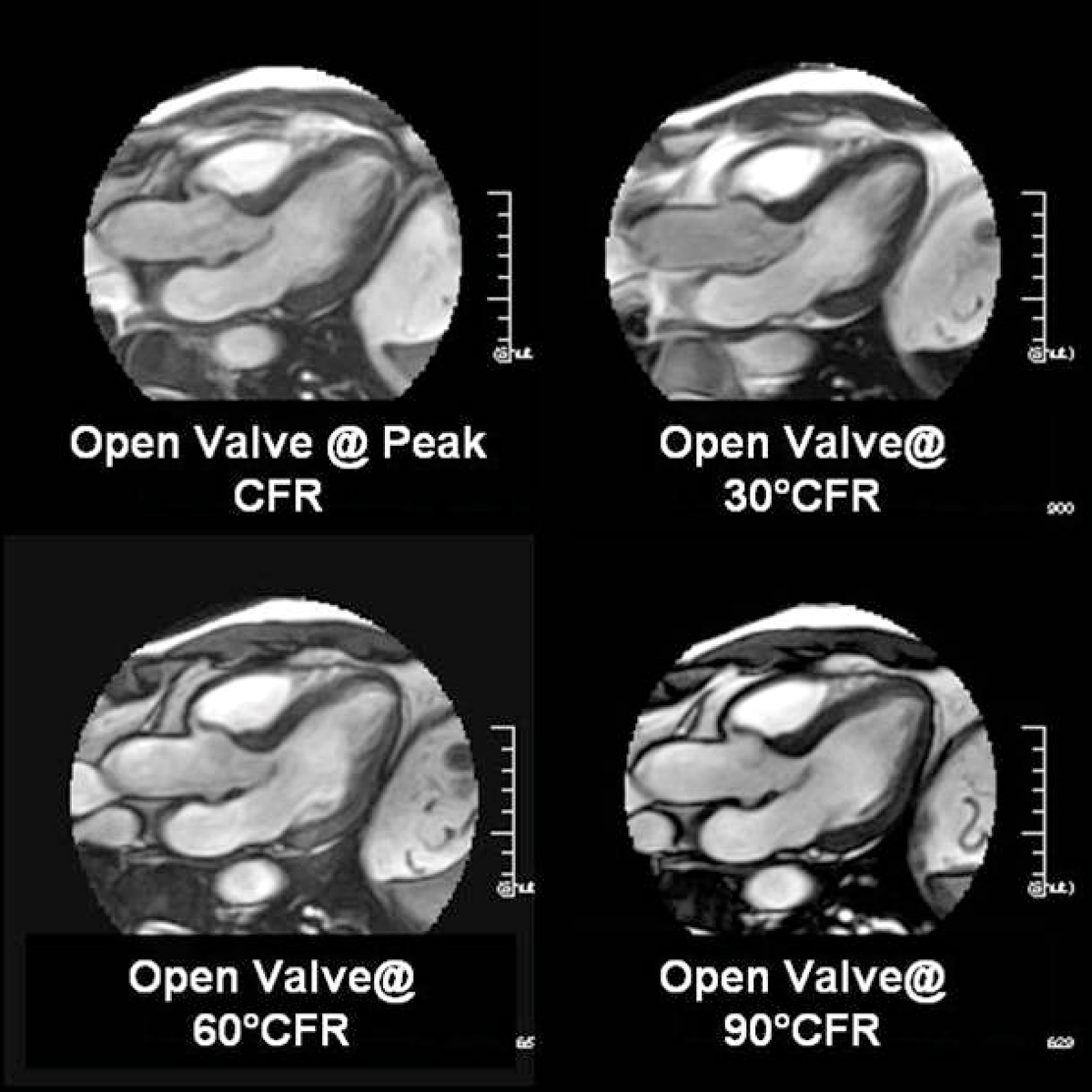
Figure 2

**Figure 3:**
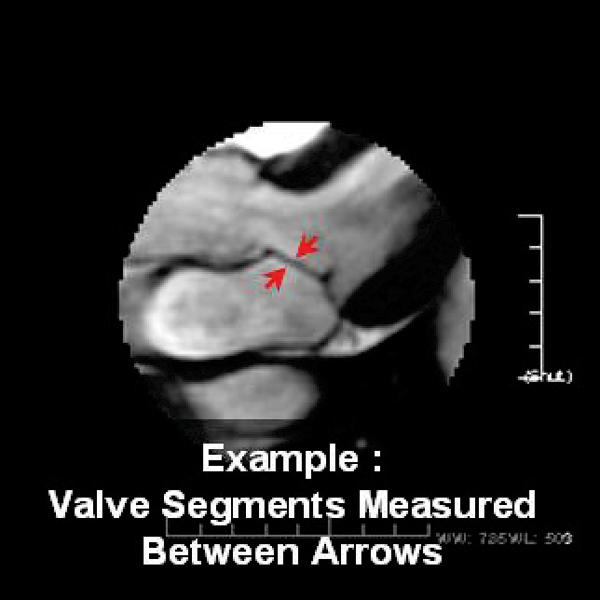
Figure 3

The specific results are summarized in Table 2. The incremental offset of the CFR, otherwise maintaining imaging parameters, allowed improved delineation of the mitral valve leaflet, and its subvalvular apparati. Despite better visualization of the valves, the measured thickness of the valves decreased when compared to the peak CFR valve measurements (approximately 5 to 12% decline in Subject A and 17 to 26% decline in subject B). We believe that this is related to the better image quality from the off resonance peak. (See Figures 1, 2, 3.)

**Table 2:** Table 2

Mitral valve leaflet measurements	Image	S1 mitral leaflet (mm)	S3 mitral leaflet (mm)	Difference between each degree offset	
				S1/S3	Comparison to Peak CFR
Subject A	Peak CFR	2.1	2.2		
	30° off peak CFR	2.0	2.1	0.1/0.1	0/30 degree
	60° off peak CFR	2.0	1.9	0.1/0.3	0/60 degree
	90° off peak CFR	2.0	1.8	0.1/0.4	0/90 degree
Subject B	Peak CFR	2.9	2.9		
	30° off peak CFR	2.4	2.4	0.5/0.5	0/30 degree
	60° off peak CFR	2.2	2.4	0.7/0.5	0/60 degree
	90° off peak CFR	2.1	2.2	0.8/0.7	0/90 degree

## Conclusion

Off resonance CFR may assist in the diagnosis of valvular abnormalities otherwise not seen clearly with routine CMR settings which utilize on peak CFR imaging. This method for valve evaluation may be beneficial when valvular and subvalvular structure evaluation is the main aim of the imaging. Unfortunately, as the frequency is shifted, the chamber flow is exaggerated therefore leading to exaggeration of valvular regurgitation or stenosis jets. Therefore the valvular jets should only be interrogated on routine SSFP imaging parameters ('On' Peak Center Frequency).

